# Surgical treatment and functional outcome of bilateral symmetrical hip dislocation and Pipkin type II femoral head fracture: a 5-year follow-up case report and literature review

**DOI:** 10.3389/fsurg.2023.1128868

**Published:** 2023-04-21

**Authors:** Sujan Shakya, Jialei Chen, Fei Xing, Zhou Xiang, Xin Duan

**Affiliations:** Department of Orthopedic Surgery, West China Hospital, Sichuan University, Chengdu, China

**Keywords:** Pipkin fracture, femoral head fracture, posterior hip dislocation, direct anterior approach Pipkin fracture

## Abstract

**Background:**

Bilateral posterior hip dislocation and femoral head fracture are rare injuries, which may be the earliest case report that focuses on treatment with open reduction via the direct anterior approach (DAA) for bilateral symmetrical Pipkin type II fracture within 5 years of the follow-up period.

**Case report:**

We present a case of bilateral posterior dislocation with a femoral head fracture (Pipkin II) of the hip in 47-year-old woman caused by a high-velocity accident. The dislocation was successfully reduced under general anesthesia at a local hospital and transferred to a tertiary center for surgical management. She was surgically treated with internal fixation using three Herbert screws on the bilateral hips. The DAA was used during surgery. Follow-up for 5 years was performed, and functional outcomes were evaluated using the D'Aubigné range of motion and modified Harris hip score. The range of motion in the bilateral hip was satisfactory, with no signs of post-traumatic arthritis, heterotopic ossification, or avascular necrosis of the femoral head.

**Conclusion:**

Surgical management of bilateral Pipkin type II fractures was performed successfully with open reduction and internal fixation using a safe and reliable direct anterior approach, with good functional outcomes at 5-year follow-up.

## Introduction

Femoral head fracture is a rare injury to the hip, accounting for approximately 4%–17% of the cases of traumatic hip dislocation cases (1). Only 1.25% of all hip dislocations are simultaneous bilateral (2, 3). Dislocations and fractures require significant forces, which are typically encountered in high-velocity motor vehicle accidents. The majority of them are either unrestrained drivers or front-seat passengers. Birkett described the first femoral head fracture followed by posterior hip dislocation in 1869 (4), whereas Garrett Pipkin classified the fracture pattern in 1957 based on the location of the head fracture in relation to the fovea and the associated lesion on the femoral head or acetabulum (5). Meislin and Zuckerman were the first to report the symmetrical bilateral Pipkin fracture type IV which was treated with cemented hip arthroplasties (6).

**Figure 1:**
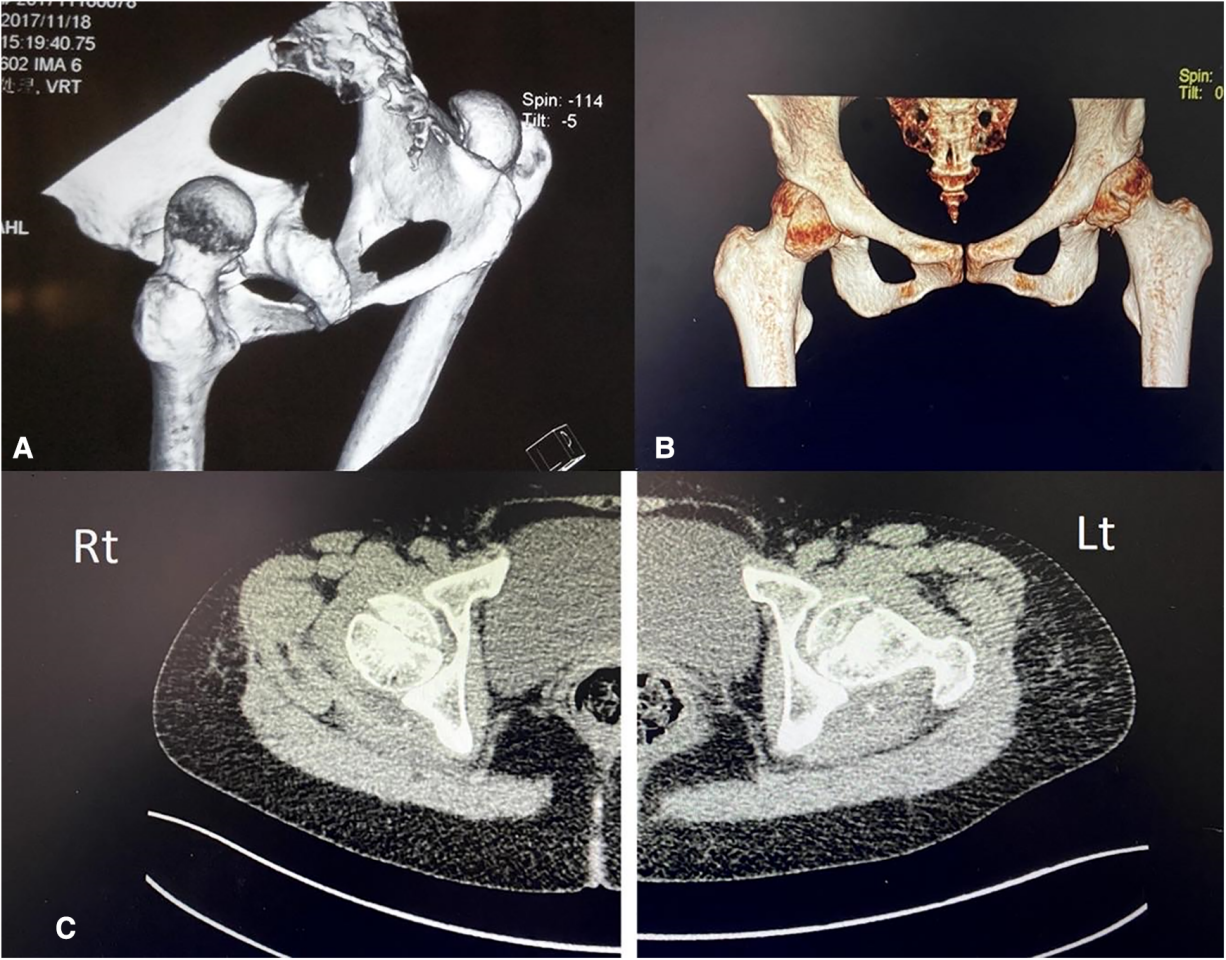
(**A**) 3D CT radiographs showing the bilateral posterior hip dislocation and femoral head fracture before the close reduction maneuver. (**B**) 3D CT radiographs showing bilateral relocated hip after reduction along with inferior part of femoral head fractures. (**C**) Plain CT transverse section of images showing bilateral intra-articular fractures of the femoral head.

**Figure 2:**
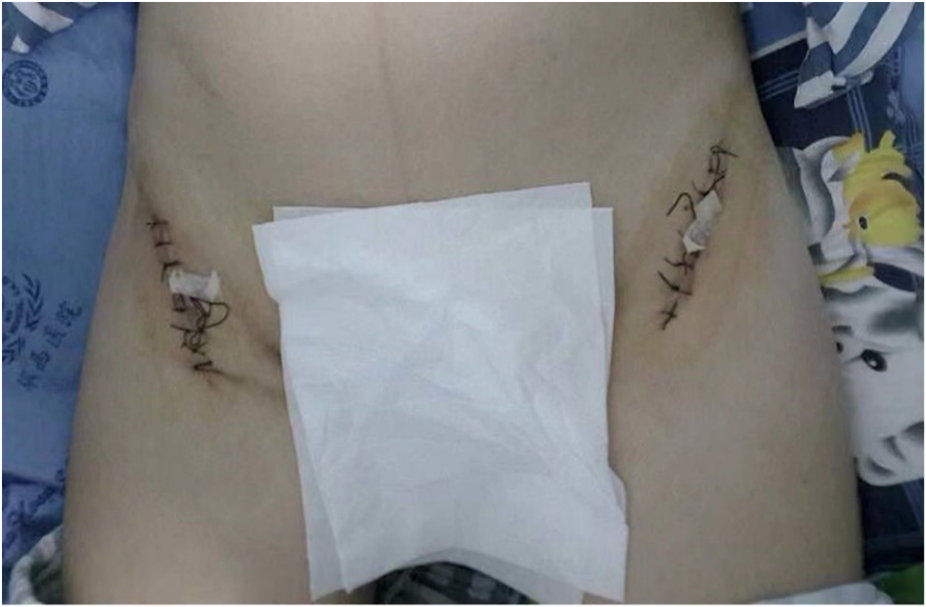
Postoperative direct anterior surgical incision over the bilateral femoral head with suture threads and rubber drainage *in situ*.

**Figure 3:**
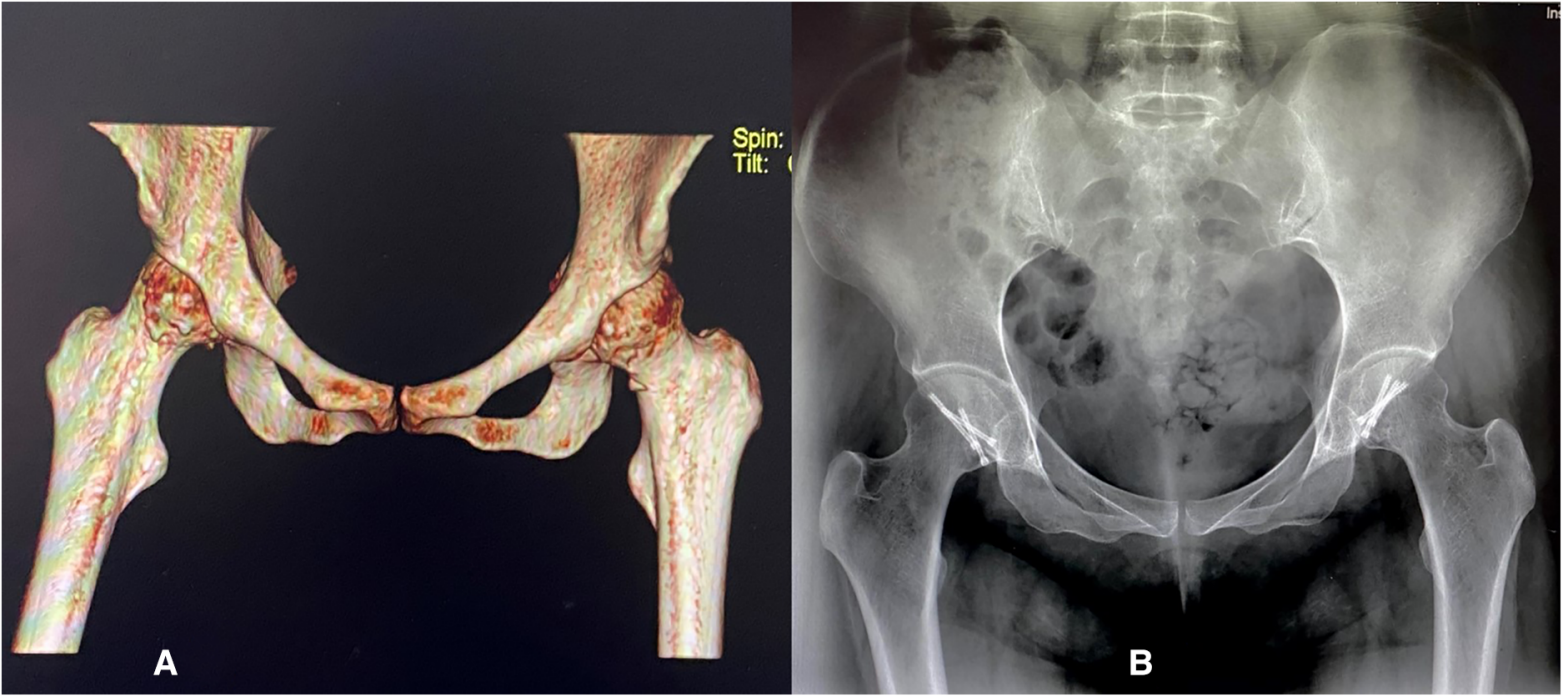
(**A**) Postoperative 3D CT and (**B**) X-ray anteroposterior (AP) images of the pelvis showing bilateral internal fixation in the femoral heads with three Herbert screws.

**Figure 4:**
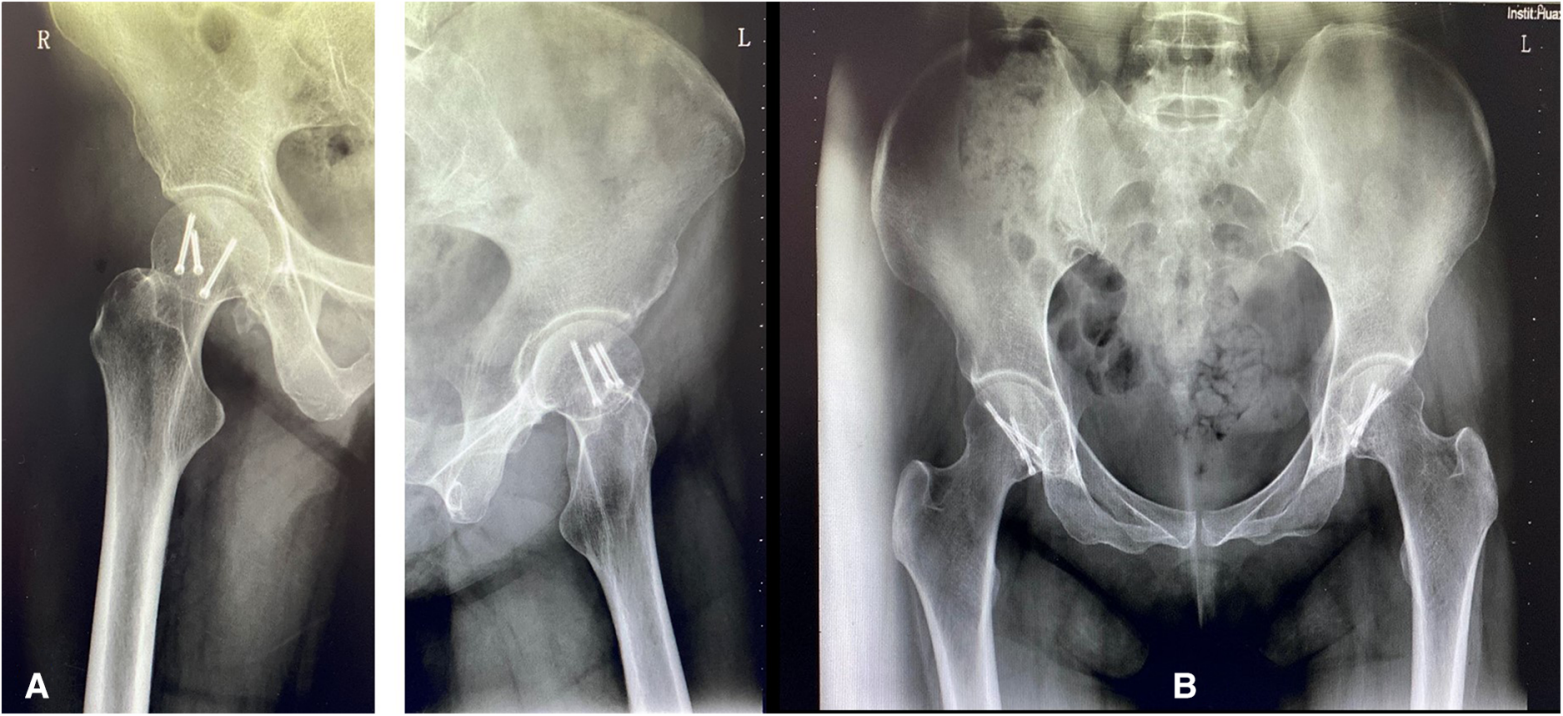
(**A**) X-ray, right and left lateral view (**B**) Plain x-ray AP view of a pelvis showing bilateral internal fixation of femoral heads during the 5-year follow-up without sign of osteonecrosis and post-traumatic osteoarthritis and heterotopic ossification.

In this paper, we report a rare case of symmetrical bilateral hip dislocation with femoral head fracture (Pipkin II) treated surgically using a direct anterior approach (DAA), with a successful outcome after a 5-year follow-up.

## Case reports

A 46-year-old woman presented with a history of a car accident 1 day ago. She complained of severe hip pain and difficulty in thigh movement. The patient presented to our hospital after the primary treatment of hip joint reduction. Due to bilateral hip pain and limited movement, radiographic examinations were performed in the emergency department of the local hospital. The patient was diagnosed with “hip dislocation and femoral head fracture.” After 1 day of inpatient observation, the patient was transported to our hospital department. The patient had no previous medical history.

### Physical examination

The patient exhibited bilateral hip tenderness and limitation of motion. No neurovascular injury was observed. She also suffered forehead collusion without loss of consciousness, lung contusion, multiple abrasions to the forehead, and lower limbs, and knee injuries. The patient was initially evaluated with advanced trauma life support protocol and stabilized hemodynamically. There was no numbness or foot drop on the bilateral leg.

### Imaging examination

Anteroposterior pelvis radiographs and 3D CT scans were rescheduled with the baseline investigation. The radiological reports suggested bilateral femoral head fracture (pipkin type II) as soon in (Figure 1), bilateral hip joint soft tissue swelling, intraarticular fluid accumulation, and mini fracture fragments.

## Treatment

### Preoperative skeletal traction

The patient underwent skeletal traction for 4 days including 1 day in the local hospital.

### Surgical procedures

On the fourth day following the incident, we decided to do surgery after a general physical assessment and anesthetic confirmation that the vital signs were stable.

During surgery, we chose the supine position for the DAA method to expose the hip. A surface projection of 2–3 cm lateral and distal to the anterior superior iliac spine was drawn, along with a 10-cm incision that is directed toward the ipsilateral femoral head. To avoid injury to the lateral femoral cutaneous nerve and femoral vessels, the tensor fasciae latae (TFL) was retracted laterally, and the sartorius muscle was retracted medially. T-shaped capsulotomy was performed to release the anterior capsule. The fractured area of the femoral head was fully exposed. The hip joint was then dislocated bilaterally, exposing the femoral head fracture while remaining in external rotation. The femoral head fragments were then reduced under direct vision. Following that, three compressive Herbert screws were installed perpendicular to the fracture line. All the screw heads were buried under the cartilage. Tiny fractured fragments and blood clots within the articulating surface of the hip were removed and washed with normal saline. Rubber drains were placed after closing the capsule (Figure 2). The total amount of bleeding for both sides was about 50 mL in 90 min. The rubber drainage was removed on the first postoperative day. On the second day, foot dorsiflexion and static quadriceps strengthening exercises were initiated immediately in bed, and postoperative radiographs were obtained (Figure 3). Celecoxib 200 mg BD (two times per day) was regularly used in clinical settings for 3 weeks to treat pain and avoid heterotopic ossification. One week later, joint flexion and extension exercises were performed. The patient was discharged from the hospital on oral medication and physiotherapy. She had no wound infections or lateral femoral cutaneous nerve injuries. The patient was advised to rest in the supine position.

## Outcome and follow-ups

One month after the operation, the patient was advised to perform partial weight bearing with the help of a walker to get out of bed. Eight months after the operation, the patient walked normally with full weight bearing without limping and normal return to daily activity. Follow-ups in first and second years showed improved modified hip score 74 (fair) and 83 (good), respectively.

According to the D'Aubigne range of motion scale, the latest hip score in the fifth year was 86 (good). The patient had a full range of movement in both hips: flexion of 120°, internal rotation of 30°, external rotation of 50°, and abduction of extension of 40°. At five-year follow-up X-rays, there was no incidence of osteonecrosis, heterotopic ossification, or signs of post-traumatic arthritis (Figure 4). She resumed daily and normal activities without any disability after a severe tragic incident.

## Discussion

Bilateral posterior dislocation of the hip occurs in approximately 1.25% of all hip dislocations (3, 7). Of posterior dislocations, 5–15% are associated with femoral head fractures (8). High-velocity motor accidents are the main cause of dislocations associated with femoral head fractures. Management of these cases is rare and controversial, and few cases have been reported in the literature. Previous studies have focused on the initial close reduction and conservative treatment. Authors such as Guiral et al. (9), and Treacy and Grigoris (10), advocated conservative treatment with skeletal traction with satisfactory outcomes. Whereas Zuckerman 1989 performed the surgical treatment with bilateral cemented total hip replacement (6). Table 1 consists of a list of English language publications retrieved from PubMed, Embase, and Google Scholar databases for the oldest available case reports and abstracts from 1962 to 2022 for bilateral femoral head fractures (Pipkin fracture). Of these, only 10 were relevant after screening every article and case report, and one case report was excluded due to the unavailability of the abstract or an entire report.

**Table:** 

Study	Year	Age/sex	Injury cause	Pipkin type	Treatment type	Approach	Outcome	Follow-up year	Complications
Kuhn et al. (11)	1987	19/female	Motor accident	Lt-IV	Lt-operative IF with screws	Osbornel	Good	1 year	–
				Rt-I	Rt-osteochondral fragment removal	Posterior			
						Approach			
Meislin et al. (6)	1989	63/female	Motor accident	B/L-IV	Cemented THA	Postero-lateral	Good	2 years	Paget's
									Disease
									HO II ^O^
Guiral et al. (9)	1992	23/female	Motor accident	B/L-II	Nonoperative	–	Good	1 year	–
Treacy et al. (10)	2002	29/female	Motor Accident	B/L-I	Nonoperative	–	Good	2 years	–
Kozin et.al. (12)	1994	71/female	Motor Accident	B/L-II, 35% femoral head fracture	Operative	Posterior approach	Good	3 years	Mild subtalar pain
					Bipolar end prosthetic replacement				
Pascarella et al. (13)	2008	23/female	Motor accident	Rt-I	Bilaterally remove the intra-articular fragments	Kocher-Langenbach posterior approach	Good	1.5 years	Lt side limitation of rotation, often painful
	Case 1			Lt-II					
Matĕjka et al. (14)	2015	—	Motor accident	Lt-II	Lt-nonoperative		Fair	18 months	Arthritis
				Rt-I	Rt-operative				
Gadi et al. (15)	2020	26/male	Motor accident	Lt-IV	–	–	–	–	–
				Rt-I					
Tripathy et al. (16)	2020	23/male	Motor accident	B/L-II	Operative with Herbert screws	Ganz's Trochanteric osteotomy	Good	2 years	–
Pathinathan et al. (17)	2021	32/male	Motor accident	Rt-I. Lt-II	Operative Lt-Herbert screws	RT-K-L approach	Good	3 months	–
					Rt-Osteochondral fragment removal				

**Table 1:** Table showing the follow-up time period and consecutive improvement in modified Harris hip score and D'Aubigne score.

No	Follow-up period	Modified Harris hip score	D'Aubigne score
1.	1 month	50	Poor
2.	1 year	74	Fair
3.	2 years	83	Good
4.	5 years	86	Good

Hip dislocation is an orthopedic emergency, and closed reduction must be performed within 6 h postinjury. The incidence of femoral head necrosis after fracture dislocation of the hip can be high, from 4.8% to 52.9%, depending on the time of reduction (18). Complications such as traumatic injuries to the sciatic nerve (mostly peroneal division), avascular necrosis of the femoral head (AVN), and post-traumatic osteoarthritis (PTOA) can occur after the incidence (19).

Only close reduction of this type of fracture pattern may also lead to AVN up to 5%–15% and may increase to 40%, requiring operative treatments (20). In our case, a closed reduction and conservative method for the larger fractured fragment on the inferior side could adversely influence the joint range of motion and stability. We also know that resection of the fragment in femoral fractures might not significantly affect the loading zone of the femoral head, but long-term consequences such as pain during mobilization and heavyweight bearing leading to arthritis as an outcome should always be considered. Therefore, we took the consideration for open reduction and anatomical internal fixation on bilateral hips.

Previously, it was considered that most internal fixation was performed using the posterior approach. The posterior approach has been associated with a greater rate of AVN than the anterior approach (21–23). Various operative approaches have been opted for Pipkin fractures depending on the femoral head fragment, the direction of the dislocation, and the associated acetabular fractures.

Several types of approaches are described in the literature: posterior (Kocher–Langenbeck), posterolateral (Moor), anterolateral (Watson Jones), trochanteric flip osteotomy, Gibson surgical hip dislocation, and direct anterior approach (Smith–Peterson) (24, 25). Our preference is for DAA, which is also interchangeably called modified Hunter’s anterior surgical approach or Smith–Peterson to treat bilateral Pipkin type II justifying the minimally invasive, adequate exposure for femoral head fixation, surgical positioning in the supine position, short hospital stays and rapid recovery of incision wounds. Our method's major assets are its least invasiveness, adequate femoral head exposure, reduced intraoperative blood loss, quicker surgery time, speedy wound healing, low visual analogue scale (VAS), and practical supine surgical position. The posterior medial femoral circumflex artery, which supplies the majority of the blood to the hip and is susceptible to damage during posterior hip dislocation, is likewise preserved by the direct anterior approach. It is interesting to note that DAA has been analyzed by a multiple researchers; the learning curve is smooth, mastery of the skills can be attained with the proper guidance, and implementation problems are rare (26). The limitation of this approach can be a contradiction in high Body Mass Index (BMI) patients. Intraoperatively, damage to the lateral femoral cutaneous nerve occurs as it passes subcutaneously between the Sartorius and TFL (26, 27).

However, this approach was related to a higher rate of heterotopic ossification (HO) (28–30). According to certain studies, HO is more common in men and elderly patients, as well as those who had primary osteoarthritis, a high body mass index, a low preoperative range of motion, and longer operations (31). We thought that low disruption of the soft tissue due to less invasive surgery, such as DAA, can lead to less local tissue damage and inflammation, especially tenotomy of the gluteus fibers and hip capsule, which may contribute to the low incidence of HO. Furthermore, pharmacological prophylaxis with selective or nonselective Nonsteroidal anti-inflammatory drugs (NSAIDs) can be used to treat HO by preventing bone formation by preventing inflammation and decreasing the osteogenic differentiation of progenitor cells (31).

Pipkin fracture is accompanied by complications such as avascular necrosis, secondary arthritis, peripheral nerve damage, and heterotrophic ossification. Nevertheless, PTOA is a long-term complication and degeneration process of the hip that can manifest as pain, decreased range of motion, and change in gait. The prevalence of PTOA following traumatic hip injury is higher in the elderly, in hip fractures with posterior acetabulum fractures, and in prolonged traumatic dislocations. Femoral head subchondral impaction or chondral injury during traumatic dislocation increases the risk of end-stage osteoarthritis leading to Total Hip Arthroplasty (THA) by 3.68 times (32). PTOA can be caused by femoral head cartilage lesions or osteochondral fragments in the joint, in addition to the quality of anatomical reduction during osteosynthesis. Even if the joint appears severely damaged on radiography, PTOA can remain silent for a long time and not manifest itself. As a result, long-term clinical monitoring and radiography are required. The radiographs of the patient was checked for signs of avascular necrosis, early sclerosis, and arthritic alterations. The clinical evaluation and radiographic assessment were correlated at the 12th week, 1-, 2-, and 5-year follow-ups.

Recently, we found many other materials, such as bioabsorbable screws, biodegradable polylactide pins, and osteochondral autografts, for the treatment of femoral head defects. Regardless, Herbert or cancellous screws are used abundantly (33). Herbert screws have the advantages of a good fixation strength and biocompatibility. It prevents the screw from protruding into the joint and has less rejection reaction that aids the fragment to part together for bone healing (34).

In summary, bilateral Pipkin fractures are rare. The key lesson to be learned from this case report is the need for immediate reduction of a dislocated hip, followed by open reduction and internal fixation using the minimally invasive direct anterior surgical technique for sufficient surgical access with a safe and effective method to treat HO, AVN, and PTOA after surgery.

## Data Availability

The raw data supporting the conclusions of this article will be made available by the authors, without undue reservation.
